# Development of a Pain Management Protocol for a Paediatric Ward in the Gambia, West Africa

**DOI:** 10.1155/2010/975313

**Published:** 2010-06-22

**Authors:** Lisa M. Puchalski Ritchie, Stephen R. C. Howie, Pamela Collier Njai

**Affiliations:** ^1^University of Toronto, University Health Network, Toronto, ON, Canada M5G 2C4; ^2^Bacterial Diseases Programme, Medical Research Council Laboratories, Fajara, P.O. box 273, Banjul, Gambia; ^3^Clinical Services Department, Medical Research Council Laboratories, Fajara, P.O. box 273, Banjul, Gambia

## Abstract

Despite recent advances in our understanding of paediatric pain and its management, pain continues to be undertreated globally, particularly in children and in low income countries. This article describes the development of a paediatric analgesia and sedation protocol, tailored to the specific setting of the Medical Research Council (MRC) paediatric ward in the Gambia, West Africa. An iterative process was used throughout development, with inputs from the medical literature, local providers, and pain experts, incorporated to ensure a safe, effective, and locally appropriate protocol. We demonstrate that evidence-based published guidelines, can and should be adapted to allow for optimal pain management given the resources and capabilities of specific health care settings. It is hoped that the process and protocol described here, will not only help to improve care on the MRC ward, but serve as an example to others working toward improving pain management in similar health care settings.

## 1. Introduction

Despite significant advances in our understanding of paediatric pain and the publication of pain management guidelines by several leading paediatric bodies in recent years, multiple studies and reviews show that pain in children continues to be poorly managed [1]. As MacLean et al. (2007) note, “there remains a gap between what we know to be effective, easily implemented pain management strategies, and what is actually practiced” [2]. Given these findings are based on their review of pain management practices in a paediatric teaching hospital in a high income country, it is perhaps not surprising this gap is larger in low income countries where resources, in their broadest sense, are more constrained.

Some barriers to managing a child's pain are common to a wide variety of health care settings, such as lack of provider training in the recognition, assessment and management of pain, and attitudes and beliefs regarding pain and its management. Other barriers likely play a significant role mainly in low income countries where resources in terms of manpower, equipment, and medications, are in chronic short supply and must be balanced across the competing needs of patients presenting to providers practicing in these settings. Despite these issues, given the current state of knowledge in this area, and the wide variety of options now available for managing paediatric pain, many of these barriers can be overcome through adaptation of published guidelines to address the unique needs and barriers of specific health care settings.

This paper describes the development of a paediatric analgesia and sedation protocol, tailored to the specific setting of the Medical Research Council (MRC) paediatric ward in the Gambia, West Africa. Although a combined paediatric and adult protocol was ultimately developed, only the paediatric portion is reported here. For clarity of presentation, the protocol development process will be described in 3 steps. However, it is important to note, that an iterative process was employed beginning with a recognized need to improve pain management expressed by local clinical staff, and with feedback from local providers sought and incorporated throughout the development process.

## 2. Methods

### 2.1. Local Consultation and Capacity Assessment

As noted above, protocol development began with an expressed desire for a pain management protocol tailored to the MRC paediatric ward, to facilitate efforts by clinical staff toward improving pain management. As a first step, key local informants including the senior clinician of the hospital, the matron, and another senior nurse, were interviewed to gain a comprehensive understanding of the hospitals resources, current practices and staff level of training, medication availability, and cost implications. Two important considerations emerged from this consultation process, which were shortage of airway support capabilities and limited staff training in pain assessment and monitoring.

To further assess the airway capabilities on the ward, a survey of equipment was undertaken. During this process, sufficient airway equipment was located to organize two complete airway kits, which included oral airways, bag-valve-masks, intubation medications and equipment. Discussions with physician and nursing staff, revealed both had little training or experience in pain management. For this reason it was felt that a more directive and structured protocol, with a strong educational component to accompany roll out of the protocol was needed.

### 2.2. Knowledge Gathering

The second step in development of the protocol began with a review of published paediatric analgesia and sedation guidelines, the evidence base for their development where possible, and consultation with experts in paediatric sedation and analgesia. As most published guidelines were developed in and for high income health care settings, during the review process and again in consultation with key local informants a list of issues relevant to paediatric pain management on the MRC ward was identified. Concerns were identified with respect to several unique attributes of the Gambian population, specifically the relative high prevalence of hemoglobinopathies [3, 4] and under-nutrition particularly in the first 2 years of life [5]. It was notable that despite the importance of cultural in perceptions and beliefs with respect to pain, no cultural issues were identified as important to pain management efforts in this setting.

Hemoglobinopathies, like sickle cell anemia and glucose-6-phosphatase deficiency (G6PD), are relatively common in the Gambia [3, 4] and present challenges for managing pain. Many established guidelines and experts recommend against sedation in sickle disease patients, and sickle trait patients with low oxygen saturations, unless anaesthesia is in attendance. Given anaesthesia support is not available at the MRC, these were included as absolute contraindications to sedation in the MRC protocol. Of additional concern is the potential for local anaesthetic induced methemoglobinemia. For a variety of reasons, including effectiveness, hemodynamic safety requiring no special monitoring, and availability of inexpensive preparations, local anaesthetics are ideal for management of brief painful procedures. While a variety of local anaesthetics have been reported to induce methemoglobinemia [6], reported cases have occurred mainly in very young infants or where excessive quantities of topical anaesthetic are used [7]. Although an uncommon complication and relatively easily managed in the general population with methylene blue, treatment of methemoglobinemia is significantly more complicated in individuals with G6PD deficiency where methylene blue can cause acute hemolysis and more intensive care, including exchange transfusions, may be required. Given the high incidence of G6PD in the Gambian population, cost and time required for testing, and potential for this serious complication whose treatment is beyond the resource capacity of the setting, use of topical anaesthetics was strictly limited to children at least 6 months of age and within recommended dosing guidelines. Guidelines and experts generally recommend 3 months of age corrected for prematurity; however, given the prevalence of under-nutrition and difficulties in accurately assessing both age of gestation and date of birth in this setting, the age restriction was broadened to ensure minimum safety guidelines were observed.

### 2.3. Protocol Circulation and Feedback

Based on the findings of the first two stages of protocol development outlined above, a draft protocol was created and circulated to key local informants and an expert in paediatric pain management for feedback and amendment. Perhaps not surprising considering the iterative nature of the development process, with the exception noted below, no major changes were suggested. At this stage the draft appeared as a numbered list of process steps with considerations at each step outlined within the section. For example, within the analgesia section, all available options were listed with their indications, contraindications, and dosages. While this format was modelled after other published protocols, and meant to emphasize options and provider choice, feedback from key local informants suggested that a more limited and algorithmic approach, presented as a flow chart would facilitate adoption of the protocol, particularly early on in the course of the campaign to improve pain management.

**Figure 1:**
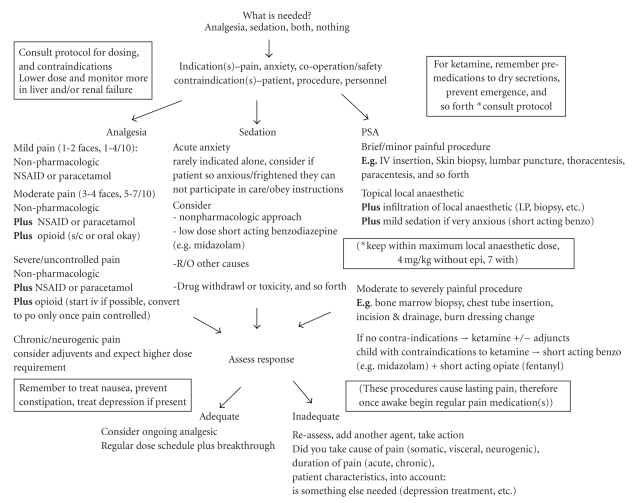
Paediatric pain management flow chart.

Based on this feedback the protocol was reworked into a flow chart (see Figure 1), with the original protocol with small amendments included as a detailed reference or guide. The amended draft was again circulated to key local informants and the pain management expert, with no further revisions suggested.

As a final step in the development of the protocol, the protocol was presented to the physician group at academic rounds, and at a staff meeting to the nursing and health attendant staff, with minor changes to the protocol incorporated as a result of feedback from these sessions.

## 3. Results and Discussion

In addition to the final product, that is, the paediatric pain management protocol tailored to the MRC ward, other benefits were gained through the process of development. As a result of the equipment survey, two airway kits were created and placed together with other basic resuscitation equipment into a strategic location within the unit for ready access.

Equally important is early evidence that through the educational and feedback sessions, further interest in improving patient care was fostered among clinical staff. As one physician reported “I encountered a case today where I would normally not have considered pain management, but after the session the other day, I decided I better do something.” As Clemmer and Spuhler (1998) have argued, “the purpose of creating protocols is greater than reducing practice variation, it also creates new paradigms and changes the culture in which health care is delivered, with the protocol itself designed to be transient, and the development process and the changes it produces, more important than the product itself” [8]. It is hoped this was only the first of many such encounters.

However, to ensure successful implementation of the protocol and ongoing improvements in paediatric pain management, further steps are needed. Ongoing education of clinical staff, particularly early in the implementation process and as new staff are hired, is essential to ensure safe and appropriate pain management procedures. Intermittent reassessment and revision based on experiences in using the protocol is needed to allow for adaptation as the needs of the patients served and the resources and options available change.

## 4. Conclusion

Despite some progress in recent years, pain continues to be undertreated globally, particularly in children [1], and particularly in low income countries [9]. Published guidelines based on best available evidence, can and should be adapted to allow for optimal pain management given the resources and capabilities of a given health care setting. It is hoped that the development process and protocol described here will not only help to improve care on the MRC ward, but serve as an example to others working toward improving pain management in similar health care settings.
